# The effects of gender and personality of robot assistants on customers’ acceptance of their service

**DOI:** 10.1007/s11628-022-00492-x

**Published:** 2022-04-26

**Authors:** Santiago Forgas-Coll, Ruben Huertas-Garcia, Antonio Andriella, Guillem Alenyà

**Affiliations:** 1 Business Department, University of Barcelona, Avda. Diagonal, 690, 08034 Barcelona, Spain; 2 Institut de Robòtica i Informàtica Industrial CSIC-UPC, C/ Llorens i Artigas 4-6, 08028 Barcelona, Spain

**Keywords:** Social-robot acceptance, Artificial intelligence, Front-office services, Gender, Robot personalities

## Abstract

The Covid-19 pandemic has stimulated the use of social robots in front-office services. However, some initial applications yielded disappointing results, as managers were unaware of the level of development of the robots’ artificial intelligence systems. This study proposes to adapt the Almere model to estimate the technological acceptance of service robots, which express their gender and personality, whilst assisting consumers. A 2 × 2 (two genders vs. two personalities) between-subjects experiment was conducted with 219 participants. Model estimation with Structural Equation Modelling confirmed seven out of eight hypotheses, and all four scenarios were estimated with Ordinary Least Squares, showing that robot gender and personality affected their technological acceptance.

## Introduction

Measures dictated to protect citizens from the Covid-19 pandemic, such as keeping social distance to avoid contact, isolation of people and patients and protective equipment for front-office staff, have contributed to boosting the use of social robots worldwide (Aymerich-Franch and Ferrer 2020; Chiang and Trimi 2020). According to Aymerich-Franch and Ferrer (2020), who analysed 195 experiences of social robot deployment in 35 countries, the main implementations were in hospitals, nursing homes, stations and airports, education centres and hospitality. The tasks they covered the most were receptionist, assistance, accompaniment and monitoring. For example, at the reception desk of hospitals, robots checked patients in upon their arrival, scheduled appointments with doctors, provided information about hospitalised patients and offered other types of indications (Aymerich-Franch and Ferrer 2020).

Social robots are programmable machines equipped with Artificial Intelligence (AI) software that allows them, on the one hand, to create the appearance of autonomy and, on the other, to exhibit social skills that facilitate human–robot interaction (HRI) (Breazeal et al. 2016). Although there is a wide variety of robotic equipment, most of the examples featured in the media are simply laboratory prototypes, and those that are commercially available (e.g. ARI, TIAGo) still have many limitations when it comes to performing complex tasks (Gale and Mochizuki 2019; PAL Robotics 2021). Thus, it is expected that in the short and medium term their use should be restricted to simple socio-emotional tasks (regardless of the cognitive-analytical complexity) and, for more complex services, they should act in conjunction with human teams (Wirtz et al. 2018). Therefore, when service managers consider implementing social robots to cover front-office tasks, they should consider the tasks to be covered and, moreover, whether the robot should act independently or in collaboration with humans.

Although the pandemic has led to the deployment of social robots, this implementation has taken place in a context of scant human resources, as staff was heavily overstretched in the health and social care sectors (Aymerich-Franch and Ferrer 2020). Yet, some applications in front-office services before the pandemic had unexpected results. For example, one of the first service companies to equip the entire establishment with robots, the Henn-na hotel in Japan, had to reinstate human staff and withdraw some of its robotic equipment due to numerous service delivery failures that contributed to a loss of productivity (Gale and Mochizuki 2019). In addition, the proposal to completely replace staff with robots was not received very well by employees or consumers (Gale and Mochizuki 2019). Other evidence, such as that collected by Pinillos et al. (2016), suggests that there has been some haste in implementing social robots to cover front-office tasks for which they were not fully equipped and this has led to some frustration and loss of confidence in their use.

Green and Viljoen (2020) saw these problems as stemming from a lack of understanding between robot designers and service managers. Whilst designers, imbued with a certain romantic view of robotics, consider AI algorithms as neutral elements and simply focus on measuring their performance in terms of how efficiently and accurately they perform certain tasks, customers and managers of service companies, influenced by a media view of robots, misinterpret their limited functions as malfunctions (Green and Viljoen 2020; Wirtz et al. 2018). Therefore, there is a need to build bridges to achieve a better understanding of both groups. Robot designers need to be more aware of market characteristics by incorporating the study of customer needs in the process of developing AI algorithms and, moreover, managers and service customers should also be aware of the limited functions of the robots on the market and thus prevent misinterpretations.

One characteristic of social robots is that they generate a completely different service experience from other self-service technologies implemented to date (e.g. ATMs [automated teller machines], airport check-in machines), as their physical appearance (non-mechanoid: humanoid or android form) and the possibility of social interaction (through their endowment of social intelligence) makes it possible to engage the customer emotionally (Van Doorn et al. 2017). However, what enables the robot to act socially is social intelligence systems, which are a set of algorithms and communication protocols that allow it to exhibit social behaviour (Złotowski et al. 2015) and enable the robot to engage consumers socially during HRI in a more meaningful way (Kim et al. 2021; Van Doorn et al. 2017). Designs for androids (human-like robots) were initially proposed, but the mismatch between their human appearance and their limited functional and social capabilities led Mori to propose the concept of the “uncanny valley”. According to his proposal, when humans interact with androids, behaviour and reactions similar to those of humans are expected, but when these do not occur because of their limited functions, feelings of unease arise (Mori et al. 2012). This has led to some consensus amongst manufacturers to propose humanoid designs (showing their mechanical nature or simplified human forms) more in line with their actual abilities and thus prevent consumer dissatisfaction (Mende et al. 2019). The trend of designing humanoids therefore seems to be the dominant one, whilst research is progressing both in incorporating new functionalities and in developing AI algorithms to improve the performance of social robots (Mende et al. 2019; Puntoni et al. 2021).

To date, human reactions to robots incorporating AI and social intelligence algorithms have been studied mainly in the field of social robotics (examples include Nakanishi et al. 2020; Pinillos et al. 2016). However, the role of AI algorithms in robots is not very clear, as robots and algorithms are often confused and evaluated together. This way of analysing robot and AI system together has been extended by studies on the implementation of robots in front-office services (Savela et al. 2018). Therefore, it is important to be clear that the same social robot can be programmed to perform different tasks at the same time, even if they are only a few (Andriella et al. 2020) and therefore each programmed service delivery will generate a different experience (Wirtz et al. 2018). Therefore, it is relevant to study technological acceptance after a direct HRI experience, in each of the different services provided by the same social robot. Another feature of these robots is that the social intelligence protocols allow them to adopt different profiles of gender, personality, social class, etc. (Dholakia 2006). When the robot adopts gender and personality stereotypes adjusted to the service to be delivered, a strong impact on the improvement of HRI is achieved (Muscanell and Guadagno 2012; Nomura 2017).

Therefore, we formulate the following research questions:

### RQ1:

To what extent does endowing a robot programmed to deliver a front-office service with gender and personality affect drivers of customers’ intention to use it?

### RQ2:

 To what extent does stereotypical consistency between the gender and personality assigned to the robot and the front-office service task to be covered affect drivers and customers’ intention to use it?

This work has three objectives. The first is to shed light on the concept of social intelligence algorithms as part of an AI system, describing the basic elements that compose them and illustrating their operation through an application. The second is to validate a technological acceptance model derived from a parsimonious adaptation of the Almere model (Heerink et al. 2010), which was the first to be used in social robotics. The third, based on the theory of fluency and previous studies (Ghazali et al. 2020), is to explore whether applying the expression of gender (female versus male) and personality (collaborative versus competitive) to social intelligence protocols in robots delivering front-office services can improve their technological acceptance.

The rest of the article is organised as follows. First, we present the conceptual framework by introducing social intelligence protocols, as well as the role of robots in front-office services and a review of the theoretical framework underpinning the different models of technological acceptance of AI-equipped humanoid robots. We then describe the role of gender and personality in social robots. Third, we describe the methodology, which consists of a pre-test, an experiment and its analysis with SEM (structural equation modelling) and OLS (ordinary least squares). Fourth, we present the results. Finally, we draw and discuss conclusions, as well as future proposals and limitations.

## Conceptual framework and hypotheses development

### Social intelligence protocols in social robotics

The AI algorithms installed in robots operate through three subsystems: a data collection and storage subsystem, a data processing subsystem (using statistical and computational applications) and finally a response subsystem (Agrawal et al. 2018). The joint action of the three subsystems creates the appearance that the robot is acting intelligently and making autonomous decisions (Puntoni et al. 2021). Each subsystem can use one or more devices that are executed in a coordinated manner. For example, Nakanishi et al. (2020) implemented and tested an AI system with two social robots (Sota and CommU) that greeted guests as they passed through a hotel lobby, attempting to replicate “omotenashi” (Japanese-style hospitality). To gather information consisting in the fact that a guest was approaching, they used a three-dimensional (3D) image sensor that detected their presence with a maximum range of 10 m. Once the presence of the host had been detected, the processing subsystem estimated the time it took for the host to reach the robots and sent a signal to the robots. Finally, the response subsystem, once the signal had been received, acted with the text-to-speech software to trigger a response consisting in greeting the guest (Nakanishi et al. 2020).

More developed social robots (e.g. ARI, TIAGo) use additional social intelligence protocols to achieve friendlier interactions with humans (Złotowski et al. 2015). For example, when a customer asks a robot for information, instead of responding aseptically, it can initiate the conversation with a greeting, it can also give some advice, offer other information (Kim et al. 2016) and even deliver messages of encouragement when trying to perform a difficult task (Fox et al. 2015). All these messages can be accompanied by facial expressions, gestures and signs that complement verbal language to create an endearing experience (Złotowski et al. 2015). However, although significant progress has been made in the development of social intelligence protocols, they still lack the ability to display verbal and non-verbal cues in an understandable, natural and enduring way (Andriella et al. 2020).

Another way in which the robot can contribute to facilitating HRI is by manifesting its own gender and even its own personality (Hwang et al. 2013). Early studies showed that adding gender to robots (by changing their physical design or voice with communication protocols) influenced both their persuasiveness and their perceived suitability for certain tasks (Eyssel and Hegel 2012; Tay et al. 2014). However, designing female robots to perform domestic tasks and male robots for surveillance and security work (Weber 2005) has been criticised for reinforcing gender stereotypes that are not acceptable in modern societies (Nomura 2017; Schiebinger et al. 2019). Although less controversial, endowing the robot with personality traits has also been proposed and shown to affect user preferences (Tapus et al. 2008).

### Social robots in front-office services

The emergence of Covid-19 has triggered the deployment of service robots (most of which are basically mechanoid) in hospitals, nursing homes, stations and airports, educational centres and universities and hospitality, amongst others, but fewer than half were equipped with social intelligence protocols allowing them to talk to users (Aymerich-Franch and Ferrer 2020; Chiang and Trimi 2020).

Front-office services produce experiences that involve not only the result of generating solutions to problems (functional element), but also delivery with high provider–customer interaction (including socio-emotional and relational elements) (Kim et al. 2021; Lee and Lee 2020; Wirtz et al. 2018). Furthermore, it is considered that, although the core of the service produces functional outcomes, what actually generates added value is the way in which the service is delivered and thus the attention received by the customer (Balanche et al. 2020; Lee and Lee 2020; Wirtz et al. 2018). Since social robotics studies HRIs (Weber 2005; Nakanishi et al. 2020), this makes it an area of knowledge closely related to service management and, more importantly, to the implementation of social robots in service companies.

For HRIs to generate memorable interactions, the robot must display a humanoid appearance and autonomy in its service delivery (Van Doorn et al. 2017). In addition, service delivery must include instrumental support (helping to solve problems), emotional support (expressing feelings of compassion in adverse situations and happiness in favourable situations) and relational support (derived from the degree of emotional bonding and trust in the interaction) (Balanche et al. 2020; Gelbrich et al. 2021; Lee and Lee 2020; Wirtz et al. 2018). On the other hand, the duration of the HRI experience also plays an important role. Thus, in short first one-off encounters, customers tend to perceive humanoid robots as if they were other people, without perceiving that they are dealing with machines (Van Doorn et al. 2017), but if the duration is longer, the perception about their true social skills is more realistic and technological acceptance is more objective (Gessl et al. 2019).

Conducting experiments with humanoid robots equipped with AI systems and social intelligence protocols is not straightforward. Commercially available robots come with very generic applications as standard, but to perform specific front-office tasks (e.g. helping a customer complete a money transfer with the ATM), they need to be programmed autonomously and installed in the robot. If, in addition, it has to greet the customer and say encouraging phrases, etc., this also has to be programmed and added as a social intelligence protocol, complemented by text-to-speech software and gesturing (non-verbal language) applications. Finally, all programmes must act in unison in order to appear realistic (Andriella et al. 2020; Nakanishi et al. 2020; Złotowski et al. 2015).

### Technological acceptance of social robots

To study the factors that explain the acceptance of new technological products (computers, smartphones, etc.) in social contexts, service marketing researchers have used models derived from social psychology. One of the pioneers, Davis (1989), proposed the Technology Acceptance Model (TAM) based on one of the most influential theories at the time, the Theory of Reasoned Action developed by Fishbein and Ajzen (1975). More than a decade later, after advances and improvements, such as TAM-2 and TAM-3, Venkatesh et al. (2003) proposed the Unified Theory of Acceptance and Use of Technology (UTAUT) model, which represented an adaptation of the Theory of Planned Behaviour (Ajzen 1991).

The UTAUT model has been extended to all new technologies (Savela et al. 2018; Wirtz et al. 2018) and its first application to social robots with social intelligence protocols was proposed by Heerink et al. (2010), who adapted a UTAUT model, which they called the Almere model, and validated it with a sample of elderly people in nursing homes. To recreate the social intelligence protocol, which allowed conversation with four robotic teams (each group with a different one, such as an iCat or a RoboCare), they used a scenario called Wizard of Oz (WoZ). WoZ scenarios, which are common in social robotics, simulate a conversation between the robot and the human, but in fact the robot is operated by a researcher from a monitor, so it is not a real experience (Ghazali et al. 2020). A decade later, Ghazali et al. (2020), considering that the technology was too new to use such sophisticated models as those derived from UTAUT, proposed that it was better to go back to the initial TAM model with the SociBot robot, albeit using the WoZ configuration. Yet, all proposals for models to study social robots consider modifications of the original TAM and UTAUT models, and the main argument for these modifications is that the humanoid embodiment and apparent social skills endow the robots with a nature that differs from that of other new technology devices (laptops or smartphones) (Ghazali et al. 2020; Heerink et al. 2010).

However, the use of both WoZ scenarios and text, images or video descriptions of social robots and their abilities, commonly found in marketing (Mende et al. 2019), have been criticised because they convey the impression that AI systems have reached degrees of sophistication far removed from reality, generating false sensations about robots’ actual abilities (Savela et al. 2018). In fact, the results of evaluations of direct experiences with robots are different to those obtained from indirect ones (WoZ, images, videos, etc.), with the former yielding more extreme and less ambivalent results than the latter (Savela et al. 2018). Therefore, it is recommended to conduct studies with real robots in order to obtain more objective results (Złotowski et al. 2015).

### Proposed model and hypotheses

To assess technological acceptance, a parsimonious adaptation of the Almere model was proposed, which pioneered the study of social robots with social intelligence protocols (Heerink et al. 2010) and, in addition, contained factors for each of the essential elements of front-office service, that is, functional, socio-emotional and relational (Balanche et al. 2020; Lee and Lee 2020; Wirtz et al. 2018). However, in line with the criticism made by Ghazali et al. (2020) of the use of extensive models, such as the Almere model, which includes six direct factors (Social Influence, Attitude, Perceived Usefulness, Perceived Ease of Use, Perceived Enjoyment and Trust) and four indirect factors (Perceived Adaptability, Anxiety, Social Presence and Perceived Sociability), we have proposed a parsimonious adaptation in which two direct drivers, Attitude and Trust, and two indirect drivers, Anxiety and Social Presence, were discarded.

First, the Attitude construct was discarded. In the first version of the TAM model, Davis (1989) proposed that internal beliefs (Perceived Ease of Use and Usefulness) were precedents of Attitude, whereas, in later versions, internal beliefs were considered direct precedents of Intention to Use by eliminating Attitude (Venkatesh et al. 2003; Zhong et al. 2020). Therefore, the fact that the Almere model considers Attitude in parallel to internal beliefs as antecedents of intention to use would indicate that it is being measured twice.

Secondly, the trust factor and the indirect factors, anxiety and social presence, have been discarded. The first two were not taken into account due to the characteristics of the robot, the participants and the public environment in which the HRI was developed (Mende et al. 2019). On one hand, humanoid robots generate the most trust and the least anxiety (Mathur and Reichling 2016). For example, anxiety, which is an intense emotional response to repressed conflict or low expectations of efficacy, is relieved by conversation and the presence of other people (Gerrig 2014). Furthermore, according to Gerrig (2014), individuals tend to show different degrees of sensitivity to anxieties, which means that this factor could play a moderating role. On the other hand, whilst in a care context where the robot replaces the nurse in a private setting, levels of trust and anxiety may be relevant, they become less important in a public setting where participants are volunteers (most likely those with lower sensitivity to anxiety) and the presence of other people reduces the perception of danger to their integrity (Mende et al. 2019). Furthermore, in the event that anxiety is relevant, given that participants are faced with a difficult task, it would be difficult to discern whether variations in anxiety levels are due to the difficulty of the task or the presence of the robot. Likewise, perceived Social Presence was not taken into consideration because its position in the technology acceptance model has not yet been clarified. Whilst Social Presence is one of the objectives pursued by social robotics (Van Doorn et al. 2017), Heerink et al. (2010) considered it a precedent of entertainment, whilst in the sRAM model it was considered a direct effect (Wirtz et al. 2018) and could even play a moderating role, since different robotic devices could represent different degrees of social presence.

**Fig. 1:**
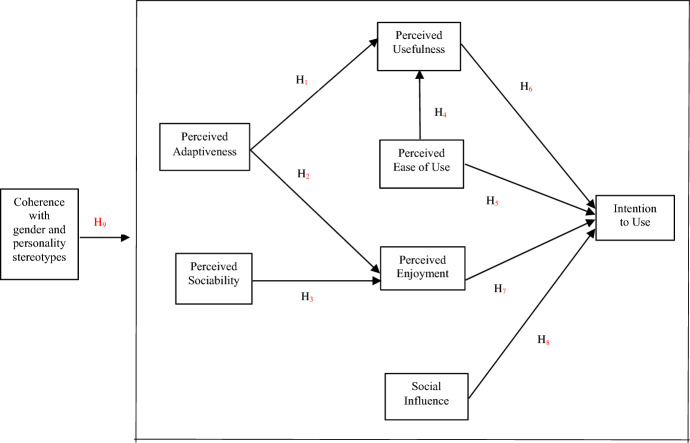
Research model

Taking into account all the proposed changes, the model consists of four direct and two indirect precedents. The model is shown in Fig. 1, and the hypothesised relationships to be validated related to assistive robots are the following:

#### H1

Perceived Adaptability is positively related to Perceived Usefulness.

#### H2

Perceived Adaptability is positively related to Perceived Enjoyment.

#### H3

Perceived Sociability is positively related to Perceived Enjoyment.

#### H4

Perceived Ease of Use is positively related to Perceived Usefulness.

#### H5

Perceived Ease of Use is positively related to Intention to Use.

#### H6

Perceived Usefulness is positively related to Intention to Use.

#### H7

Perceived Enjoyment is positively related to Intention to Use.

#### H8

Social Influence is positively related to Intention to Use.

### The impact of gender and personality stereotypes on social robot acceptance

Social intelligence protocols allow robots to be endowed with human attributes, such as gender, personality and cultural level, although the most prominent have been gender and personality (Dholakia 2006; Muscanell and Guadagno 2012; Nomura 2017).

Stereotypes are inaccurate beliefs about qualities or abilities shared by certain groups of people or collectives, which contribute to setting expectations about their behaviour (Tay et al. 2014). One of the theories that explain the existence of stereotypes is the theory of fluency, which proposes that when subjects are repeatedly exposed to a stimulus, it is stored in memory, so that the next time they re-experience it, it will familiar to them and they will process it more easily (Whittlesea 1993). Designing robots to appear feminine or masculine is very easy, as any gender attribution, such as the tone of voice, already triggers users to believe that it is a male or female robot (Dryer 1999; Tay et al. 2014). Evidence has been published showing that gender stereotypes make it easier for HRIs to be more intuitive (Eyssel and Hegel 2012), improve users’ attitude (Tay et al. 2014) and increase persuasiveness and the perception that they perform their tasks appropriately (Eyssel and Hegel 2012). On the other hand, as with humans, when there is a mismatch between gender and the task performed by the robot, a bias against it is activated, leading to a negative evaluation (Jarman et al. 2012).

However, their use has been criticised for reproducing stereotypes that are reprehensible in modern societies (Nomura 2017; Robertson 2010). Moreover, dissenting voices such as Dufour and Nihan (2016) indicate that the incorporation of gender stereotypes in robots is not necessary to improve their acceptance or their economic value. This study is proposed from scientific realism, which implies trying to perform the analysis in a way that is as aseptic as possible (Hunt and Hansen 2009). However, in line with Schiebinger et al. (2019), we consider that robots are designed in a world dominated by gender norms, identities and relationships that are not going to change in the short term and therefore, pragmatically, we must accept that this is the context in which such studies take place and, what is more important, that they give rise to debate.

Alongside gender, personality is another key aspect in the generation of intuitive responses during HRI (Lee et al. 2006). Indeed, it shapes the nature of social relationships and influences the level of satisfaction derived from them (Dryer 1999). Although addressed to a lesser extent than gender, previous studies have shown that endowing the robot with personality influences users’ preferences (Canal et al. 2019), perceived enjoyment, perceived intelligence and attractiveness (Lee et al. 2006). However, providing the robot with personality is more complex than endowing it with gender due to the multiple dimensions that shape human personality. For example, Dryer (1999) manipulated both the voice and sentences of an artificial agent (Chatbot) to configure two personality traits: extraversion (at both extremes: extraverted–reserved) and agreeableness (cooperative–competitive) and tested how this affected human reactions. But, in addition, Weber (2005) established that there is a correspondence between personality traits and gender stereotypes. That is, in the agreeableness trait, females are often characterised as more cooperative (indicating communality), whereas males are often characterised as more competitive (indicating agency) (Eyssel & Hegel, 2012). Furthermore, as with gender stereotypes, if the stereotypical behaviour is violated, transgressors will be evaluated more negatively (Jarman et al. 2012).

In this study, social intelligence protocols will reproduce gender–personality attributes in social robots and study their effect on the technological acceptance of a robot performing a front-office task considered feminine (Weber 2005). We expect that when the robot acts as a male or a female and with a cooperative or competitive personality trait, conceptual fluency will cause users to recognise these as human and to act accordingly. Thus, we predict that when the robot’s gender and personality match the task according to the stereotypical belief, it will create a positive conceptual fluency experience that will enhance its technological acceptance more than when the gender and personality do not match the stereotypical assigned task (Weber 2005). Based on the above information, the following hypothesis is proposed:

#### H9

Robots that show conceptual stereotypical gender and personality coherence with the assigned task (e.g. a female–cooperative robot performing help and assistance tasks) will have a more positive influence on the robot’s technological acceptance than when there is a mismatch (e.g. a male–competitive robot performing help and assistance tasks).

## Methodology

To test and validate the proposed hypotheses and try to answer the research questions, we conducted an experiment with a robot equipped with an AI system and four social intelligence response protocols. On reviewing previous studies, we discovered that, in order to obtain a sufficiently large sample in real establishments, for example, a hotel, we would need an amount of time that was beyond our reach. For example, Nakanishi et al. (2020) needed two weeks in a hotel corridor to collect 67 evaluations from the Sota and CommU robots, and Pinillos et al. (2016) needed two months with the Sacarino robot in a hotel lobby to record 349 experiences. So, instead of setting up the robot in a hotel or hospital, we decided to simulate an equivalent task in terms of time, help requirements and robot attention that would allow us to collect a large sample in a short time. To do so, we set up a booth at a trade fair that is organised every year in the city, visited by thousands of people, where we invited the attendees to participate in a game assisted by a social robot. Since this study aims to validate a model composed of seven scales (26 items), it will require a sample size of at least about 200 subjects, between 5 and 10 cases per item (see Kline 2011, pp. 11–12). Simulation experiments, as in this study, are used when real-life marketing policy evaluation could be too complicated, time consuming or prohibitively expensive (Tkachenko et al. 2016). Nevertheless, as Wolfe and Roberts (1993) pointed out, they can yield similar results to those obtained in field experiments, i.e. achieve comparable external validity.

### Experiment

A between-subject 2 (robot gender: male vs. female) × 2 (robot personality: cooperative vs. competitive) experiment was proposed involving the recreation of a scenario in which a robot equipped with an AI system helps customers to complete a difficult task. To recreate the service, we drew on the examples of experiences with self-service machines (ATM, airport check-in machines, etc.) proposed by Meuter et al. (2005), where customers who must follow and complete a sequence of commands often get stuck, and on Solichin et al. (2019), who studied hotel reception tasks and observed that this activity involves a first contact with the guest and an interaction of about four minutes, during which the receptionist greets the guest, asks how the trip went, asks for documentation and helps to fill in forms, etc. Based on this information, an AI system was designed, with functions similar to those of completing a sequence of commands and with a degree of emotional and relational bonding similar to that of receptionists, as well as being capable of maintaining a conversation for about five minutes (Andriella et al. 2020).

**Fig. 2:**
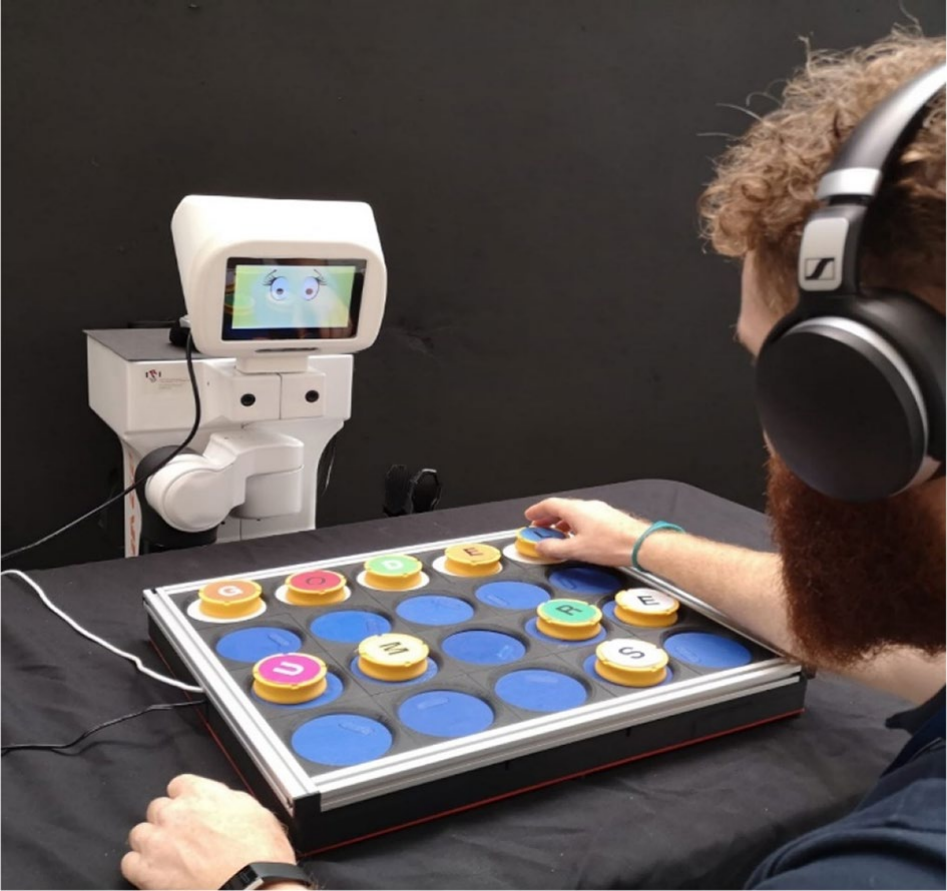
Participant playing the game with the assistance of the robot

As a simulator of a scripting task, we proposed a board game consisting in trying to complete the five-letter name of a Nobel laureate (e.g. “CURIE”) from ten letter tokens (Fig. 2 shows an image of the game) (Andriella et al. 2020). Furthermore, to recreate an emotional bond, the robot assisting the player offers advice on where to find the correct token (Kim et al. 2016), as well as messages of encouragement depending on how the task is being completed (Fox et al. 2015).

### Robotic equipment

A TIAGo robot, equipped with an AI system to manage the game and four responsive subsystems, was used as a service provider, assisting participants as they completed the game. The TIAGo is a highly versatile semi-humanoid robot that combines perception, navigation, manipulation and HRI skills and also has one of the fastest and most efficient processors on the market (NVIDIA® Jetson™ TX2), which allows the programming of functional activities, as well as social communication protocols and the coordination of social functions (PAL Robotics 2021).

A programmed AI system was incorporated into the robot to complete the three stages. To collect the data, a board and tokens with electronic terminals were used to detect any movement of the tokens, information that was stored and, in addition, also served to activate the information processing subsystem. The movement of each token (right or wrong) and the time between movements were processed and, in turn, activated the response subsystem (Agrawal et al. 2018). The response subsystem used four social intelligence protocols that aimed to represent a female/cooperative, male/cooperative, female/competitive and male/competitive robot by means of verbal and non-verbal signals. The verbal signals were reproduced by the text-to-speech software Loquendo (which uses a male and female voice to reproduce the programmed Catalan text). The script was structured in three parts: (1) Introduction, a researcher activates the game and the robot begins by introducing the game and the type of help it is going to offer; (2) During the game, the AI system counts the time it takes the participant to move a token and triggers the issuing of advice on where to look – if s/he picks up the wrong token, it issues messages expressing discouragement like “Mmmmh”, “Really?” and “Are you sure?” and if s/he picks up the correct token, it issues encouraging messages such as “Great”, “Yes” and “Wow”; and (3) Farewell, when the participant has completed the game, the robot emits a goodbye message. For non-verbal language, the original head was replaced by one with a built-in Liquid Crystal Display screen, and graphic design software was used to recreate cartoon-type eye expressions. The joint performance of the three subsystems (collection, processing and response) generates the perception that the TIAGo robot acts intelligently and makes autonomous decisions (Puntoni et al. 2021). Figure 2 shows a picture of a volunteer playing with the robot.

To recreate the gender, we manipulated the tone of voice, using either a male or a female voice, as well as appropriate facial features (long eyelashes for the female robot and a moustache for the male). And, to recreate personality, we manipulated the tone of the script for the conversation with expressions of praise and support to represent the cooperative role and more challenging expressions to represent the competitive role, in a similar way to what has previously been done in the literature (Andriella et al. 2020; Canal et al. 2019; Tay et al. 2014). Table 1 shows the conversation translated into English.

**Table 1:** Social conversation protocols according to robot personality

Challenging robot	Praise robot	Action	Timing
This is a puzzle game. You need to find the name of an inventor using the tokens available on the board. You need to start from left to right. Do not try to break the rules. During the game I can provide you with assistance if you're very bad. Remember, you can move the token only when I say it's your turn. I expect this will be a trivial game for you	This is a puzzle game. You need to find the name of an inventor using the tokens available on the board. You need to start from left to right. During the game I can provide you with assistance. Remember, you can move the token only when I say it’s your turn	Instruction	Before starting
It is a trivial game and you took longer than expected	It has been a pleasure playing with you, hope you enjoyed it too	Instruction	At the end
The guy before you was performing better, try to be more concentrated	Come on… I know you can do it	LEV_1	Before user move
Really? Can’t you do better than this?	I believe in you!!	LEV_1	Before user move
So far, not so promising	Don’t be afraid to make a mistake	LEV_1	Before user move
You really need more assistance? Look on the right	I’ll give you a clue, look on the right	LEV_2	
You really need more assistance? Look at the centre	I’ll give you a clue, look at the centre	LEV_2	
You really need more assistance? Look on the left	I’ll give you a clue, look on the left	LEV_2	
No, no…	Mmmhhh, are you sure?	Wrong token	Pick
Really, you cannot fail	Nope	Wrong token	Pick
Very bad	Try a different one	Wrong token	Pick
Come on, really? That’s so easy, I don’t know how to help you	No worries, it happens sometimes	Wrong token	Place
I don’t understand what you’re doing	I know how you feel – I’ve been in this situation before	Wrong token	Place
Really? That’s completely wrong	People say lucky in love, unlucky at play	Wrong token	Place
Trivial	Wow, you found it	Correct token	Pick
OK	Great	Correct token	Pick
OK, but too slow	Yes	Correct token	Pick
I expect more from you	Well done, you’re playing as I expected	Correct token	Place
Well, you can do better	You’re so good	Correct token	Place
That’s the best you can do?	Congratulations, that’s the correct letter	Correct token	Place
It’s your turn	It’s your turn	Instruction	–
You have to follow my rules, move the tokens back now	Move the token back please	Unexpected action	Place

### Pre-test

A pre-test was conducted to verify whether the manipulation of gender and personality were perceived differently. A sample of 21 Master of Business Administration students (female: 76%, mean age: 32 years) were randomly assigned to one of two experimental conditions (female–collaborative and male–challenging robot), where they evaluated a video showing a TIAGo robot playing a board game with a person. The robot’s perceived gender and personality were measured by rating six items on a five-point scale (1 = “strongly disagree” and 5 = “strongly agree”): “The robot expresses itself as masculine (feminine)”, “The robot seems competitive (collaborative)” and “The robot seems challenging (flattering (you receive praise))”. The results of an analysis of variance (ANOVA) showed that gender manipulation was easily identified. When the robot played a male role, it was described as more masculine (*Mean (M)* = 4.45, *standard deviation (SD)* = 1.03) than feminine (*M* = 1.50, *SD* = 0.45; *F* (1.21) = 66.89*, p* < 0.01) and, vice versa, when it played a female role, it was said to be less masculine *(M* = 1.63, *SD* = 1.20) than feminine (*M* = 4.41, *SD* = 0.79; *F* (1, 21) = 43.40, *p* < 0.01). Although the two personalities were also identified, they did so in a less forceful way. Thus, the cooperative robot was rated as more collaborative (*M* = 3.90, *SD* = 1.51) than competitive (*M* = 2.41, *SD* = 1.24; *F* (1, 21) = 6.74, *p* < 0.05) and, vice versa, the competitive robot was seen as more competitive (*M* = 4.25, *SD* = 0.62) than collaborative (*M* = 2.81, *SD* = 1.53; *F* (1, 21) = 8.86, *p* < 0.01). And finally, participants judged the competitive robot as more challenging (*M* = 3.90, *SD* = 1.09) than flattering (*M* = 2.58, *SD* = 1.31; *F* (1, 21) = 7.10, *p* < 0.05) and, vice versa, the cooperative one was described as more flattering (*M* = 3.83, *SD* = 1.02) than challenging (*M* = 1.81, *SD* = 1.16; *F* (1, 21) = 19.34, *p* < 0.01). The manipulation was therefore perceived correctly.

### Participants and procedure of the main study

We set up a stand at a trade fair for new technologies and sustainable products, which attracts thousands of visitors to Barcelona every year and recruited 219 participants (Table 2 shows demographic data).

**Table 2:** Respondents’ profile

Variable	Demographic characteristics	Frequency	Percentage
Sex	Male	113	51.6
	Female	106	48.4
Age	18–24 years	42	19.2
	25–34 years	66	30.1
	35–44 years	43	19.6
	45–54 years	41	18.7
	Above 54 years	27	12.4
Nationality	Spanish	183	83.6
	Others	36	16.4

The procedure consisted of three stages. The first was recruitment based on gender quotas. We invited participants to complete a game with the help of a robot. We read them their rights, they gave their permission and we gave them a brief explanation of the game (estimated time: 5 min). Second, in batches of ten participants per scenario, where we controlled for gender, they completed the board game with the help of one of the TIAGo robot profiles (5 min). Third, the participants answered the questionnaire consisting of 26 statements that had to be evaluated using a five-point Likert-type scale (1 = “strongly disagree” and 5 = “strongly agree”) and the identification data. Given that the scales taken from the literature are in English, the back-translation method proposed by Brislin (1970) was used for their translation into Spanish. This method is based on three phases: first, the original scales are translated into Spanish; second, they are revised and retranslated back into English; and third, the two versions (the original and the retranslated) are compared and revised, and possible divergences are corrected. Three linguists (two bilingual researchers and a professional translator) were involved in the process. Table 3 presents the constructs and items used, as well as the source from which they were adapted. The technological acceptance model of the robot equipped with an AI system was validated with a structural equation model (SEM) based on variance and covariance matrices, by maximum likelihood with EQS 6.4 and with the ML Robust estimation method to avoid normality problems (Bentler 2006). Moreover, the four response subsystem scenarios, given the size of each cell, were adjusted with OLS. The proposed model is shown in Fig. 1.

**Table 3:** Constructs and items used

Code	Construct	Items		Adapted from
ITU	Intention to Use		(1) If the robot was available, I would try to use it	Palau-Saumell et al. (2019)
			(2) If the robot was available, I would try to use it whenever I could in my spare time	
			(3) If the robot was available, I would sometimes think about when I could use it	
PU	Perceived Usefulness		(1) I think the robot is useful for entertaining	Heerink et al. (2010)
			(2) It would be nice to have the robot for entertaining	
			(3) I think the robot could be used to entertain me and do other things	
PEOU	Perceived Ease of Use		(1) I immediately learned how to use the robot	
			(2)The robot seemed easy to use	
			(3) I think I can use the robot without any help	
			(4) I think I can use the robot with someone’s help	
			(5) I think I can use the robot if I have some good instructions	
PENJ	Perceived Enjoyment		(1) It’s fun to talk to the robot	
			(2) It’s fun to play with the robot	
			(3) The robot looks enjoyable	
			(4) The robot seems charming	
			(5) The robot seems boring	
SI	Social Influence		(1) I think my friends would like me to use the robot	Heerink et al. (2010); Pujadas-Hostench et al. (2019)
			(2) I think it would give a good impression if I played with the robot	
			(3) I think that people whose opinion I value would be pleased to see me play with the robot	
PAD	Perceived Adaptiveness		(1) I think the robot could adapt to my needs	Heerink et al. (2010)
			(2) I think the robot would adapt to what I need at each moment in the game	
			(3) I think the robot will help me when I consider it necessary	
PS	Perceived Sociability		(1) Talking to the robot is amusing	
			(2) I find the robot pleasant to interact with	
			(3) I feel the robot understands me	
			(4) I think the robot is attentive	

## Results

### Scales validation

Before testing the model, the descriptive statistics of the collected data were analysed. As the questionnaires were completed in the presence of a researcher, who was available to answer any possible questions, and were followed up in detail, none of questionnaires were incomplete. Furthermore, the values obtained were within the range of acceptability for skewness (between −3 and 3) and kurtosis (between −10 and 10) (Brown 2006). Having seen the descriptive statistics, the dimensionality, reliability and validity of the measurement scales of the constructs were analysed.

From the confirmatory factor analysis, 5 of the 26 items were eliminated due to the low loads obtained, leaving 21 items that made up the seven dimensions (three items per construct). Their measurements, depicted in Table 4, show adequate reliability and convergent validity. The constructs achieved a Cronbach’s *α* above 0.80, composite reliability (CR) higher than 0.80 (ranging from 0.83 to 0.94) and all the items exhibited adequate convergent validity. Each factor load exceeded 0.6 and the t-values for each item were significantly high according to what is recommended by the literature (Hair et al. 2010). Its discriminant validity was also ratified (Table 5). The square root of the average variance extracted (AVE) between each pair of factors was larger than their estimated between-factor correlations, which means that any construct must share more variance with its items than with the other constructs in the model (Fornell and Larcker 1981).

**Table 4:** Psychometric properties of the measures

Items	Factor loading	T	Mean	Standard deviation	Skew	Kurtosis
*Intention to use*						
(Alpha: 0.92; AVE: 0.82; CR: 0.92)						
ITU1	0.95	22.75	2.46	1.21	0.45	− 0.65
ITU2	0.89	19.54	2.74	1.22	0.27	− 0.8
ITU3	0.83	13.92	2.18	1.15	0.8	− 0.14
*Perceived usefulness*						
(Alpha: 0.87; AVE: 0.74; CR: 0.88)						
PU1	0.76	11.71	3.87	1.24	− 0.9	− 0.22
PU2	0.94	19.90	3.65	1.23	− 0.6	− 0.58
PU3	0.83	16.57	3.38	1.21	− 0.46	− 0.6
*Perceived ease of use*						
(Alpha: 0.82; AVE: 0.67; CR: 0.83)						
PEOU1	0.84	13.11	3.93	1.14	−0.86	−0.25
PEOU2	0.81	11.37	4.1	1.06	−1.14	0.63
PEOU3	0.71	12.48	3.71	1.19	−0.61	−0.65
*Perceived enjoyment*						
(Alpha: 0.82; AVE: 0.68; CR: 0.84)						
PENJ3	0.93	21.82	3.53	1.37	−0.54	−0.96
PENJ4	0.82	17.81	2.91	1.32	0.01	−1.14
PENJ5	0.61	9.48	3.7	1.18	−0.57	−0.63
*Social influence*						
(Alpha: 0.93; AVE: 0.84; CR: 0.93)						
SI1	0.98	24.87	3	1.18	−0.11	−0.78
SI2	0.94	22.51	2.9	1.19	0	−0.79
SI3	0.79	16.18	3.12	1.22	−0.14	−0.87
*Perceived adaptiveness*						
(Alpha: 0.85; AVE: 0.71; CR: 0.86)						
PAD1	0.74	14.42	3.01	1.25	0.01	−1.03
PAD2	0.86	16.43	3.1	1.12	0.01	−0.7
PAD3	0.85	16.27	3.28	1.22	−0.22	−0.85
*Perceived sociability*						
(Alpha: 0.86; AVE: 0.74; CR: 0.88)						
PS2	0.96	25.88	3.02	1.28	−0.06	−1.09
PS3	0.81	15.22	2.71	1.24	0.23	−0.92
PS4	0.73	11.35	3.42	1.2	−0.51	−0.68

The model fits Chi-squared (*χ*^*2*^): 174.4986; *df:* 167; *p*: 0. 32,972*; RMSEA*: 0. 014; *CFI*: 0.998 and *NNFI*: 0.997

*AVE* is the average variance extracted, *CR* is the composite reliability

**Table 5:** Discriminant validity of the scales

	ITU	PU	PEOU	PENJ	PSI	PAD	PS
ITU	0.90						
PU	0.58***	0.86					
PEOU	0.03 ns	0.35***	0.82				
PENJ	0.45***	0.54***	0.37***	0.83			
PSI	0.57***	0.47***	0.07 ns	0.40***	0.92		
PAD	0.57***	0.62***	0.32***	0.57***	0.55***	0.84	
PSI	0.34***	0.35***	0.26***	0.54***	0.30***	0.51***	0.86

Below the diagonal: correlation estimated between the factors

Diagonal: square root of AVE

*p < 0.05; **p < 0.01; ***p < 0.001

### Model analysis

**Fig. 3:**
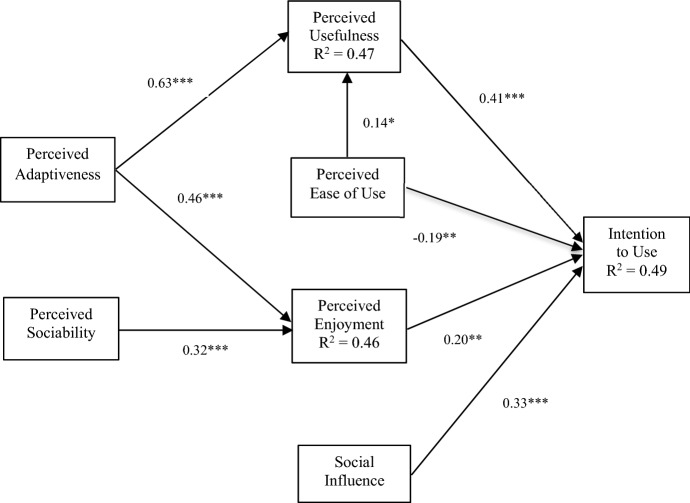
General structural model results (*p < 0.05; **p < 0.01; ***p < 0.001)

The intention-to-use model of the robot equipped with an AI system (including the four response subsystems) was fitted using SEM and achieved acceptable *R*^*2*^ values for the sample size (Hair et al. 2010): 0.49 for Intention to Use, 0.47 for Perceived Usefulness and 0.46 for Perceived Enjoyment (see Table 6 and Fig. 3). For example, in similar experiments where aggregated values of experiences with different robots were analysed, although with results that are not directly comparable, Turja et al. (2020) reached a pseudo-*R*^*2*^ value of 0.285, whilst Heerink et al. (2010) provided the *R*^*2*^ of the partial models of each robot (ranging from 0.49 to 0.79), but does not provide the *R*^*2*^ obtained with the path analysis of the global model.

**Table 6:** Causal relations in the general model

Independent variable	Dependent variable	Beta	T	R^2^
PU	ITU	0.41	5.39	0.49
PEOU		− 0.19	− 2.83	
PENJ		0.20	3.16	
SI		0.33	5.47	
PEOU	PU	0.14	1.98	0.47
PAD		0.63	7.20	
PAD	PENJ	0.46	5.70	0.46
PS		0.32	3.75

From Fig. 3, it appears that the Intention to Use a robot equipped with an AI system, after receiving its assistance to complete a complicated task similar to a front-office service, was mainly explained by the Perceived Usefulness (*β* = 0.41, *p* < 0.05), by the Social Influence that the environment generated on participants (*β* = 0.33, *p* < 0.05) and by Perceived Enjoyment, indicating that they had a good time (*β* = 0.20, *p* < 0.05), thus supporting H6, H7 and H8. However, the degree of Ease of Use had a negative effect on Intention to Use. At the same time, Perceived Usefulness was mainly described by Perceived Adaptability to the visitor (*β* = 0.63, *p* < 0.05) and, in this case, also by Ease of Use (*β* = 0.14, *p* < 0.05), thereby supporting H1 and H4. In addition, the feeling that interacting with a robot seems entertaining, i.e. Perceived Enjoyment, was defined by Perceived Adaptability (*β* = 0.46, *p* < 0.05) and, albeit with less intensity, by the perception of being in contact with a sociable being (Perceived Sociability) (*β* = 0.32, *p* < 0.05), thus supporting H2 and H3. Considering that seven of the eight hypotheses have been confirmed and, with the same sign as the original Almere model, most of this parsimonious adaptation of the original model has been validated.

To test H9, the sample was divided into the four response subsystems, which configured the combination of gender and personality, giving subsamples of 55 participants in Scenario 1 (S1), 58 participants in S2, 52 participants in S3 and 54 participants in S4. From each subsample, the parsimonious adaptation of the Almere model was estimated using OLS (Table 7 shows the results obtained). Starting with the stereotypically consistent scenarios: S1 (female-cooperative robot) explained the highest proportion of the Intention to Use a social robot, reaching an *R*^*2*^ = 0.62. The main direct drivers of this model were Social Influence (*β* = 0.60, *p* < 0.05) and Perceived Usefulness (*β* = 0.27, *p* < 0.05). Regarding indirect relationships, Perceived Usefulness was explained by Adaptability (*β* = 0.50, *p* < 0.05) and Perceived Enjoyment was also explained by Adaptability (*β* = 0.32, *p* < 0.05) and Perceived Sociability (*β* = 0.28, *p* < 0.05). On the other side of the coin, the totally incoherent scenario S4 (male–competitive robot) explained the model to a lesser extent (*R*^*2*^ = 0.41). Three factors, Perceived Usefulness (*β* = 0.48, *p* < 0.05), Social Influence (*β* = 0.31, *p* < 0.05) and Ease of Use, described the Intention to Use, although negatively in the case of the latter (*β* = -0.28, *p* < 0.05). And, albeit indirectly, Perceived Usefulness was explained by Adaptability (*β* = 0.58, *p* < 0.05) and Ease of Use (*β* = 0.24, *p* < 0.05), whilst Perceived Enjoyment was also explained by Adaptability (*β* = 0.55, *p* < 0.05).

**Table 7:** Causal relations in SCENARIOS 1,2,3,4

Independent variable	Dependent variable	Scenario 1 Beta	T	R^2^	Scenario 2 Beta	T	R^2^	Scenario 3 Beta	T	R^2^	Scenario 4 Beta	T	R^2^
PU	ITU	0.27*	2.57	0.62	0.33*	2.58	0.49	0.28*	2.08	0.50	0.48*	3.36	0.41
PEOU		−0.17	−1.88		-0.30*	-2.68		0.06	0.53		−0.28*	−2.37	
PENJ		0.07	0.68		0.44*	3.72		0.20	1.49		−0.03	−0.21	
SI		0.60*	5.89		0.17	1.35		0.37*	3.07		0.31*	2.44	
PEOU	PU	0.12	1.03	0.29	0.18	1.42	0.25	0.17	1.66	0.52	0.24*	2.27	0.43
PAD		0.50*	4.16		0.40*	3.13		0.65*	6.31		0.58*	5.41	
PAD	PENJ	0.32*	2.47	0.25	0.35*	2.83	0.29	0.26*	2.22	0.54	0.55*	4.17	0.41
PS		0.28*	2.15		0.31*	2.54		0.56*	4.76		0.14	1.11	

Significant at *p < 0.05

As for the rest of the scenarios, S2 (male–cooperative robot) achieved an *R*^*2*^ value of 49%. This dependent variable was directly explained by Perceived Usefulness (*β* = 0.33, *p* < 0.05) and Perceived Enjoyment (*β* = 0.44, *p* < 0.05), but negatively in the case of the Ease of Use (*β* = -0.33, *p* < 0.05). Moreover, Perceived Usefulness was explained only by Adaptability (*β* = 0.40, *p* < 0.05) and the fact that it seemed enjoyable was explained by Adaptability (*β* = 0.35, *p* < 0.05) and Perceived Sociability (*β* = 0.31, *p* < 0.05). Finally, in S3 (female–competitive robot) the model explained 50% of the variance in the intention to use a social robot. In this case, it is mainly explained by Social Influence (*β* = 0.34, *p* < 0.05) and Perceived Usefulness (*β* = 0.28, *p* < 0.05). At the same time, Perceived Usefulness was explained by Adaptability (*β* = 0.65, *p* < 0.05) and, furthermore, Perceived Enjoyment was defined by Perceived Sociability (*β* = 0.56, *p* < 0.05) and by Adaptability (*β* = 0.26, *p* < 0.05).

**Fig. 4:**
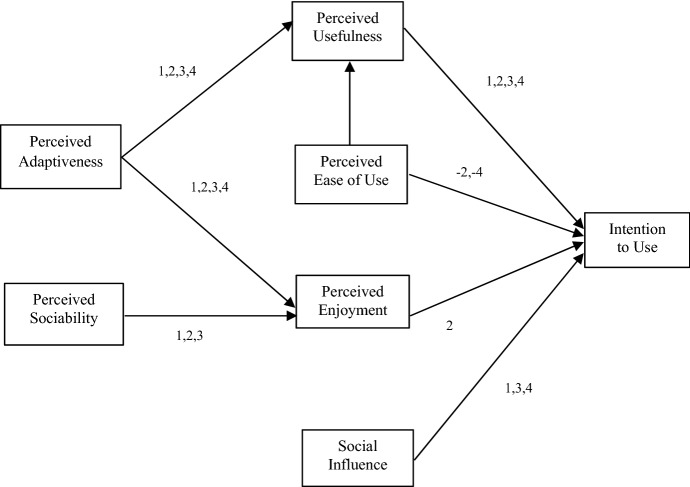
Model results. Significant causal relationships of each scenario. 1 = Female/Cooperative, 2 = Male/Cooperative, 3 = Female/Competitive, 4 = Male/Competitive

All this evidence has helped to answer the research questions. On the one hand, the robot’s gender and personality have affected model fit and the main drivers that explain intention to use. On the other hand, regarding the second question, whether stereotypic consistency between gender and personality and assigned service task improves model fit and drivers, the answer is less conclusive. Thus, the robots that played the role according to the assigned task stereotype achieved greater fit than the totally unmatched one (*R*^*2*^ robot S1 – *R*^*2*^ robot S4 = 0.21). When applying the Fisher transformation and estimating the difference in correlations (*z* = 1.608, *p* < 0.05), it was concluded that this difference was significantly higher for the female–cooperative robot than for the male–competitive one. However, although S1 (female–cooperative robot) is the one that best fitted the proposed model, according to the Fisher transformation there were no significant differences with the other scenarios, with S2 (male–cooperative robot) or with S3 (competitive–female robot). This difference could be considered as a measure of the moderating effect of gender–personality interaction on the type of task performed and its effect on the intention to use a social robot (Hayes 2018). Thus, H9 would be partially corroborated, as the female–cooperative role explains the model to a greater extent in front-office service tasks than the male–competitive role, but not with the other combinations (Eyssel and Hegel 2012). However, the findings indicated that for front-office service tasks, gender plays a somewhat more important role than personality in achieving conceptual coherence of stereotypes. The OLS results of the four scenarios are shown in Table 7, and Fig. 4 summarises the significant relationships.

## Discussion, conclusions and limitations

The aim of this research has been to shed some light on the concept of a social intelligence algorithm by understanding the AI systems installed in social robots in order to provide front-office customer services. Although social robots started their journey in front-office service delivery a couple of decades ago, some early applications have yielded discouraging results (Gale and Mochizuki 2019). We believe that these implementations were carried out without a clear understanding of the nature of the service experience (which should incorporate both analytical-cognitive and socio-emotional elements) or of the current imbalance between the first and second elements of commercialised robots (Chiang and Trimi 2020; Wirtz et al. 2018).

Furthermore, to illustrate the functioning of AI systems and responsive subsystems, an experiment was conducted with a social robot simulating a front-office service, and its technological acceptance was estimated. Findings support a validation of the parsimonious version of the Almere model for estimating the technological acceptance of a semi-humanoid robot with an AI system, since seven of the hypotheses considered have reached significant values and with the expected sign. Finally, it was found that applying gender (feminine versus masculine) and personality (collaborative versus competitive) expressions to the response subsystem may contribute to greater acceptance in tasks consistent with their stereotype. Next, we discuss the theoretical and managerial implications of our findings.

### Theoretical implications

A more parsimonious version of the Almere model has been proposed and validated with a sample of visitors to a trade fair, who evaluated a TIAGo robot equipped with an AI system and four response protocols. This adaptation was performed due to both a theoretical controversy, as in the case of the factors Attitude and Social Presence, and context, because of the type of robot used (a humanoid) and the environment in which the experiment was conducted (voluntary participation and a public space) (Wirtz et al. 2018). In addition, the use of robots equipped with real AI systems to assess consumer perceptions has been advocated, as WoZ scenarios or the use of videos often show degrees of sophistication outside reality that led to incorrect perceptions (Mende et al. 2019; Złotowski et al. 2015).

The lack of standardisation and the fact that in the Almere model four different types of robotic equipment were assessed, whilst in our study the same robot used four output subsystems does not allow the findings to be compared directly. However, their comparison can help to indicate the degree of consistency of the results (Wirtz et al. 2018). In our adaptation, the fundamental antecedents of the intention to use are the perception that it is a useful tool, its social influence and the fact that it is entertaining. These factors were also found to be significant in the work of Heerink et al. (2010), albeit with different weights, thereby suggesting that HRIs are affected by the type of service offered and the situational context (Lin et al. 2020; Nakanishi et al. 2020). However, participants’ lack of familiarity with them makes it difficult to appreciate whether they are easy or difficult to use, and this could explain the negative effect of ease of use on the intention to use, although they are able to recognise its effect on perceived usefulness. Similar results on the acceptance of social robots were found in Taiwanese restaurants (Lee et al. 2018) and hotel services (Lin et al. 2020), although in these models perceived ease of use had an indirect, rather than a direct, effect on acceptance. Heerink et al. (2010) obtained the opposite result, where ease of use was one of the main precedents of intention to use and, in the case of functional robots in hospitality, ease of use was the main driver of perceived value (De Kervenoael et al. 2020).

In addition, after interacting with the robot, players perceived that it adapts to their needs and influences the perceived usefulness and, in turn, this adaptability, together with the perception that it behaves as a social agent leads to enjoyment. Heerink et al. (2010) found similar effects between adaptability and utility, as well as between sociability and enjoyment. The relationship between sociability and enjoyment is connected to the basic objectives of social intelligence, which consist in transmitting not only a simple response but also an emotional state (Lazzeri et al. 2013), and both scenarios fulfil them.

On the other hand, it has been considered that social intelligence protocols adapted to gender and personality expressions in the response subsystem can play a relevant role. Fluency theory predicts that people playing a stereotypical role would improve their acceptance (Eyssel and Hegel 2012) and the same results have been obtained with social robots. This proposal to assign “feminine” tasks to female robots has been criticised because it contributes to perpetuating gender stereotypes in society (Nomura 2017; Robertson 2010). This study follows a scientific realism approach (Hunt and Hansen 2009) and, in line with Schiebinger et al. (2019), posits that in the short term this situation will not change and cannot be stopped from developing.

The results show that two profiles, out of the four resulting from combining gender and personality, explained a greater variance of intention to use the robot than in the overall model, thereby suggesting a certain moderating effect according to RQ1. Thus, in the female robots, participants considered social influence the most relevant driver in explaining their intention to use, with a higher weight than perceived usefulness. In the opposite gender (the male robot), perceived usefulness is more or less important to explain intention to use, depending on their personality. These results are in line with those obtained by Heerink et al. (2010) in the Almere model, where the partial fits with each of the robotic devices differ from each other. Furthermore, the robot playing the stereotyped role appropriate to the assigned task (female–cooperative robot) achieves a higher fit than its antagonist (male–cooperative robot), pointing to a positive response to RQ2. Hence, for the male–cooperative case, entertainment is the main driver of intention to use over utility, whilst for the male–competitive, perceived usefulness is the most relevant factor over social influence. Conversely, perceived adaptability leads to a greater perception of usefulness and enjoyment in all robot profiles, whilst ease of use is a driver of perceived usefulness only for the male–competitive robot. Furthermore, perceived sociability has been one of the relevant factors to explain enjoyment in almost all robot profiles except for the male–competitive one, where it did not reach a significant value.

Findings suggest that gender and personality stereotypes work for social robots when they perform tasks in line with their profile, i.e. that perceptual fluency is activated and mental shortcuts facilitate this outcome. Nonetheless, further research is needed to corroborate these findings, as well as to address the debate about the social desirability of these results.

### Managerial implications

Companies that use social robots need to know that front-office services produce experiences that involve functional elements as well as socio-emotional and relational elements (Kim et al. 2021; Lee and Lee 2020; Wirtz et al. 2018). Although service companies have used social robots with a functional focus in easily automatable tasks, such as food preparation and serving in restaurants (De Kervenoael et al. 2020; Chiang and Trimi 2020; Lin et al. 2020), their transition to front-office settings seems more complicated, since commercialised prototypes have not yet achieved sophisticated AI systems capable of social intelligence skills that facilitate HRI in a fluid and natural way (Andriella et al. 2020).

In this research, a TIAGo robot was equipped with a social AI system with the three components, namely, a data collection and storage subsystem, an information processing subsystem and a response subsystem (Agrawal et al. 2018). In addition, four response subsystems have been tested by simulating their performance as an assistant, offering advice and empathetic feedback to users when faced with a difficult task, and thanks to this help all participants managed to finish their task correctly. That is, the social robot can adapt its social AI to guide users to successfully complete a sequence of commands, e.g. with ATMs, ticket vending machines and check-in machines (Meuter et al. 2005). In addition, the degree of acceptance of a robot’s profiles (combination of gender and personality) may change depending on the task it performs (care of the elderly, security or entertainment).

### Limitations

This research has some limitations. First, for the sake of convenience, a sample of visitors at a trade fair and a board game have been used, instead of a sample of guests completing some front-office tasks at a hospital or hotel reception. However, this experience has provided us with a lot of information to improve social intelligence protocols. Therefore, future research could examine more gender–personality combinations with different tasks and determine whether the support provided improves robot acceptance and even increases clients’ persistence in completing the task (Gelbrich et al. 2021).

The model developed in this study captures a short first-time HRI, and the findings reflect that experience. Therefore, we can consider that as familiarity with robots increases, and the model should be adapted to the new reality. Moreover, a convenience sample formed by people who agreed to participate in the study was used, so it is not possible to make any statistical inferences. However, it is worth noting that the sample was fairly balanced between men and women and covered a wide range of ages.
